# Construction and effect evaluation of prediction model for red blood cell transfusion requirement in cesarean section based on artificial intelligence

**DOI:** 10.1186/s12911-023-02286-1

**Published:** 2023-10-12

**Authors:** Hang Chen, Bowei Cao, Jiangcun Yang, He Ren, Xingqiu Xia, Xiaowen Zhang, Wei Yan, Xiaodan Liang, Chen Li

**Affiliations:** 1 School of Computer Science and Technology, Xi’an Jiaotong University, Xi’an, 710049 Shaanxi China; 2 Department of Information Service, Shaanxi Provincial People’s Hospital, Xi’an, 710068 Shaanxi China; 3 Department of Transfusion Medicine, Shaanxi Provincial People’s Hospital, Xi’an, 710068 Shaanxi China; 4 Beijing HealSci Technology Co., Ltd, Beijing, 100022 China

**Keywords:** Artificial intelligence, Precise blood use model, Clinical application, Effect evaluation

## Abstract

**Objectives:**

This study intends to build an artificial intelligence model for obstetric cesarean section surgery to evaluate the intraoperative blood transfusion volume before operation, and compare the model prediction results with the actual results to evaluate the accuracy of the artificial intelligence prediction model for intraoperative red blood cell transfusion in obstetrics. The advantages and disadvantages of intraoperative blood demand and identification of high-risk groups for blood transfusion provide data support and improvement suggestions for the realization of accurate blood management of obstetric cesarean section patients during the perioperative period.

**Methods:**

Using a machine learning algorithm, an intraoperative blood transfusion prediction model was trained. The differences between the predicted results and the actual results were compared by means of blood transfusion or not, blood transfusion volume, and blood transfusion volume targeting postoperative hemoglobin (Hb).

**Results:**

Area under curve of the model is 0.89. The accuracy of the model for blood transfusion was 96.85%. The statistical standard for the accuracy of the model blood transfusion volume is the calculation of 1U absolute error, the accuracy rate is 86.56%, and the accuracy rate of the blood transfusion population is 45.00%. In the simulation prediction results, 93.67% of the predicted and actual cases in no blood transfusion surgery; 63.45% of the same predicted blood transfusion in blood transfusion surgery, and only 20.00% of the blood transfusion volume is the same.

**Conclusions:**

In conclusion, this study used machine learning algorithm to process, analyze and predict the results of a large sample of cesarean section clinical data, and found that the important predictors of blood transfusion during cesarean section included preoperative RBC, surgical method, the site of surgery, coagulation-related indicators, and other factors. At the same time, it was found that the overall accuracy of the AI model was higher than actual blood using. Although the prediction of blood transfusion volume was not well matched with the actual blood using, the model provided a perspective of preoperative identification of high blood transfusion risks. The results can provide good auxiliary decision support for preoperative evaluation of obstetric cesarean section, and then promote the realization of accurate perioperative blood management for obstetric cesarean section patients.

## Introduction

In recent years, the number of cesarean sections has gradually increased, while obstetric hemorrhage is still the main cause of cesarean section morbidity and mortality worldwide [1]. Bleeding during cesarean section may lead to hysterectomy, maternal death and other adverse outcomes [2]. Blood transfusion is an effective emergency measure for the anemia, which may occur in cesarean section bleeding. Studies have shown that the blood transfusion rate for cesarean section in developed countries is 1.1-7.8%, and the highest in developing countries is more than 12.2% [3, 4]. According to the CDC, about 650 women die from pregnancy-related complications in the United States every year, of which obstetric hemorrhage is the leading cause of maternal death. Worldwide, hemorrhage remains the leading cause of maternal death and the most common form of shock in obstetric practice [1]. Known risk factors for bleeding during cesarean section include antenatal anemia, uterine atony and other factors [5]. But it is very difficult to accurately predict blood transfusion.

With the improvement of medical technology and the development of blood transfusion therapy, the blood transfusion strategy has gradually changed from an empirical type to a restricted and precise type. However, the prediction of intraoperative blood transfusion for cesarean section by anesthesiologists and obstetricians is mainly based on clinical experience, and there is still no objective basis for reference [6]. This often leads to an overestimation of blood requirements and an increased risk of blood disease transmission and adverse effects from blood use. The current perioperative blood management strategy adopted in obstetric cesarean section, on the one hand, wastes blood resources to a certain extent, and on the other hand, it may be difficult to identify the real high-risk groups of blood transfusion in advance. Therefore, there is an urgent need for technical means that can achieve precise blood transfusion in cesarean section operations, which can accurately predict the needs of patients for intraoperative blood transfusion, identify high-risk groups for cesarean section blood transfusion in advance, and achieve accurate patient blood management for cesarean section.

With the rapid development of information technology, the informatization work of the medical industry has made great progress. Especially in recent years, big data technology and artificial intelligence technology have gradually matured. Machine learning (ML) is a kind of artificial intelligence technology, using data to train models and use models to make predictions, which has been widely used in the medical field [7, 8]. It can help anesthesiologists make objective predictions and judgments by analyzing, calculating and predicting a large amount of clinical data [9, 10]. Machine learning models can provide more accurate predictions for patients with better predictive performance than traditional statistical methods [6, 11].

At present, some studies have applied artificial intelligence technology to the medical field, and some of them have also carried out artificial intelligence applications in the field of blood transfusion [12–16]. These studies demonstrate the feasibility of applying artificial intelligence to blood transfusion prediction. However, there are still some shortcomings in the current research. First, there are few studies and poor performance in blood transfusion prediction. The best R^2^ for the transfusion volume prediction model in the study by Andreas was 0.176 [16]. In the study by Feng [17], the transfusion volume prediction model was directly compared with the doctor’s blood preparation results, but not with the actual blood transfused volume. Second, previous studies have used blood transfusion status to represent patients’ blood transfusion needs, which is somewhat unobjective. Different doctors may affect blood transfusion due to differences in experience. Different levels of blood shortage at different times may also affect blood transfusions. Therefore, it is difficult for the blood transfusion situation in the historical data to objectively reflect the actual blood demand of the patients. Lastly, previous transfusion models were unable to provide predictive value under different transfusion strategies. The study found that there was no significant difference in the effect of restrictive blood transfusion on prognostic indicators compared with unrestricted blood transfusion. Restrictive blood transfusions may be more adopted in the future. The training model based on the blood transfusion situation in the historical data cannot meet the prediction of blood transfusion under different blood transfusion strategies in the future. Using hemoglobin as a measure, on the one hand, can objectively reflect the patient’s real blood transfusion needs, and on the other hand, it can provide predictive values for different blood transfusion strategies. When constructing the prediction model of cesarean section blood transfusion volume, further optimization needs to be done for the above deficiencies.

This study intends to construct an AI model for intraoperative red blood cell transfusion demand prediction in cesarean section surgery. Combined with postoperative hemoglobin, the model predicted results were compared with the actual results. Provide data support and improvement suggestions for the realization of accurate blood management of cesarean section patients.

## Methods

### Data sources

The elective cesarean section operation from 2017 to 2021 in the obstetrics department of a tertiary hospital was used, and the screening was performed according to the following inclusion and exclusion criteria. Inclusion criteria: (1) elective surgery for cesarean section in obstetrics; (2) application for preoperative blood preparation; (3) surgical blood for the purpose of blood use; (4) red blood cell transfusion; (5) complete medical records. Exclusion criteria: (1) non-surgical blood transfusion; (2) only non-erythrocyte transfusion blood is included; (3) key parameters such as preoperative Hb are missing, and core parameters such as preoperative hemoglobin, height, and weight are not included in these core parameters. The parameters are critical for intraoperative blood transfusion prediction, so this part of the data is excluded; (4) the evaluation parameters such as Hb after operation are missing, and the lack of this parameter will not be able to accurately evaluate the blood consumption, so this part of the data is also excluded.

We collected patient-related data from several in-hospital information systems, such as HIS, LIS, and blood distribution system, including demographic characteristics, clinical diagnosis, surgery details, blood routine, coagulation function, biochemical indexes, blood gases, and vital signs. For unstructured information such as clinical diagnosis and surgery name, we used NLP technology to split sentences into words with the smallest unit, and extracted key textual information affecting cesarean section blood transfusion from them, such as characteristics of twin fetuses, keloidal uterus, placenta previa, premature rupture of membranes, and placenta previa. Although the operation time is one of the influencing factors of intraoperative blood transfusion risk, we constructed the model with the aim of preoperative prediction of intraoperative blood transfusion needs, and therefore only included the relevant indicators that were available preoperatively, and did not incorporate variables such as the operation time, which was available only after the operation was completed. In addition, none of the patients in this study had autologous blood used during the procedure and all were transfused with homologous blood.

### Data cleaning

To ensure the high quality of the data, the collected raw data is cleaned and processed, including field mapping, data filtering, data cleaning, data replacement, data calculation, data verification, data merging, data splitting and other functions to ensure the effectiveness of subsequent models, as well as the accuracy of predictions. After applying inclusion and exclusion criteria and performing data cleansing, 4,702 individuals were removed from the initial 14,849, leaving a total of 10,147 individuals in the dataset.

### Model construction

Use the blood transfusion surgery data of the whole hospital from 2017 to 2019 to train the model, select the eligible blood transfusion surgery, and use the machine learning algorithm to train the intraoperative blood transfusion prediction model. The intraoperative blood transfusion volume was used as the outcome indicator, and the predictive variables were based on literature search and clinical experience, including patient information, test results and other indicators, and surgical information and other clinical indicators to construct an artificial intelligence model.

From 2014 to 2019, 14,849 cases of cesarean section with blood application were selected and 10,147 cases were screened for model training after passing the criteria of admission and discharge. Eighty% and 8,115 cases were selected as the training set, and 20% and 2,032 cases were used as the test set. From January 2020 to September 2021, 3,764 cesarean section operations with Shen blood were selected, and 3,255 operations that met the criteria of inclusion and exclusion were selected for simulation prediction.

Data acquisition: Blood transfusion spans a diverse and cross-disciplinary domain, necessitating the comprehensive collection of clinical data from patients. This includes fundamental patient information, admission and discharge details, surgical records, laboratory test results, vital sign readings, and any other patient-related data pertinent to blood transfusion processes. Furthermore, all data relevant to blood transfusion activities, such as blood application records, blood characteristics, and transfusion specifics, must be included. This entails aggregating disparate patient data stored across multiple databases. Ultimately, a comprehensive blood transfusion database is constructed, centered around the blood transfusion process, and organized by patients. This database encompasses all pertinent indicators associated with blood transfusion practices.

Feature engineering: There are often abnormal data in medical data due to improper recording, which will not seriously affect the model effect. Therefore, error values are generally replaced with null values during data cleaning.

For the features with different importance, different imputation processing methods are adopted. Such as surgery name, surgery diagnosis, and preoperative Hb are the most important features. If they are missing, it will seriously affect the prediction effect of the model. Therefore, if the data is missing, the record will be excluded, that is, not included in the training and test sets and simulation predictions gather. In addition, some features with a missing rate of more than 70% are also removed.

There are also some features, such as height and weight, which are also very important for indirectly assessing the blood volume of patients during modeling. These values are also seriously missing in some departments. For such demographic characteristics, according to other characteristics such as age, gender, etc., the mean value of the same category of patients in the hospital is obtained for filling. For other less important features, use − 999 uniform imputation.

It can be seen from the experience of the surgeon: the location of the operation, the surgical method, whether it is minimally invasive, the disease, medical history, imaging examination results and other factors have a great impact on the amount of blood loss and blood transfusion in the elective hand, and their importance is often higher than the biochemical and vital signs. Therefore, the processing of such textual description information is particularly important.

The text is preprocessed first to correct some common writing errors. Then, through the commonly used word segmentation technology, jieba word segmentation is used here, the sentence is divided into words with the smallest granularity, and divided into different core medical information categories, such as body parts, surgical approaches, operation methods, diseases (states), restrictions, equipment, drugs, etc. Among them, the parts are the most important, and the knowledge graph method is used to further classify the parts to form a tree structure of the parts for the next step of statistical and abstract feature extraction at different categories. At this time, the text is split into multiple core keyword combinations, which avoids the impact of word order differences and subtle differences between non-keywords.

Model method: The gradient boosting decision tree used in this study is a kind of nonlinear algorithm with very high accuracy. Using 300 to 1,000 decision trees, it can effectively capture very high-dimensional correlations, and the accuracy is much higher than that of logistic regression, Traditional models such as support vector machines. As we all know, the core of artificial intelligence is to be able to find rules from wrong predictions and make self-correction. Therefore, on this basis, this research will focus on developing machine learning models with self-learning as the core technology. Taking the accurate prediction of preoperative clinical blood use as the entry point, for those cases where the AI model prediction is wrong, but the doctor’s prediction is accurate and the blood transfusion strategy is reasonable, in addition to giving more weights for machine learning, an adaptive regularization algorithm is also proposed. Penalize the branches that misclassify the reinforcement samples and increase the proportion of this part of the pruning. Machine learning can effectively improve the accuracy of the model, learn from the experience of different doctors, and the improved model is automatically deployed in the clinic, forming a virtuous circle.

### Effect evaluation method

#### Blood transfusion or not

First compare the difference between whether the model predicts blood transfusion and whether blood is transfused. A cross-tabulation table was constructed based on actual and predicted blood transfusions, aiming to analyze the effectiveness of the proposed model.

#### Blood transfusion volume

Second, a direct comparison of the model-predicted blood transfusion volume versus the actual volume of blood transfused was performed. The overall indicators such as the actual average blood transfusion volume and the predicted average blood transfusion volume, and individual statistical indicators such as the ratio of the actual blood transfusion volume to the predicted blood transfusion volume are compared. And can be divided into departments, surgical methods to present the comparison results. Equal blood use: Actual blood transfusion volume (U) = predicted blood transfusion volume (U). Inequal blood use: Actual blood volume (U) ≠ predicted volume (U). In this study, the blood transfusion unit (U) is an integer. The precision in this comparison focuses solely on the integer component, disregarding any variations in the decimal portions.

### Targeting postoperative hemoglobin

Finally, compare blood transfusion volumes based on postoperative hemoglobin targeting (determining a postoperative Hb target range, e.g., 80–100 g/L). At the same time, for cases where the actual and the predicted are not equal, a detailed analysis of indicators such as platelets is used to judge which is more reasonable between the actual blood use and the predicted blood use.

The definition of the accuracy of actual blood use is as follows: For surgery without actual blood transfusion, if the actual postoperative hemoglobin is greater than or equal to the lower limit of the target postoperative hemoglobin (80 g/L), the actual blood use is accurate; if the actual postoperative hemoglobin is less than the target surgery After the lower limit of hemoglobin (80 g/L), the actual blood consumption is wrong, and the blood consumption is insufficient. For the actual blood transfusion operation, if the actual postoperative hemoglobin is greater than or equal to the target postoperative hemoglobin lower limit (80 g/L) and less than or equal to the target postoperative hemoglobin upper limit (100 g/L), the actual blood use is accurate; if the actual postoperative hemoglobin is less than the target postoperative hemoglobin lower limit (80 g/L) or greater than the target postoperative hemoglobin upper limit (100 g/L), the actual blood use is error. For the operation with wrong actual blood use, it can be further divided into actual blood use excessive or actual blood use insufficient. The specific definition is shown in the table below. The actual blood usage accuracy rate is: actual blood usage accurate operation times/total operation times * 100%.

### Statistical analysis

Measurement data are expressed by Mean (SD), Median (P25, P75), and count data are expressed by frequency (percentage). For the comparison of the indicators between the transfusion group and the non-transfusion group, the independent t test or Mann-Whitney U test was used for continuous variables, and the χ2 test or Fisher test was used for categorical variables. The model uses indicators such as AUC for model evaluation.

## Result

### Baseline setting

From 2014 to 2019, 14,849 cases of cesarean section with application for preoperative blood preparation were selected and 10,147 cases were screened for model training after passing the criteria of inclusion and exclusion. The cesarean section data from January 2020 to September 2021 were selected for simulation prediction, and 3,255 operations were included. The demographic characteristics and blood transfusion status of obstetric cesarean section patients are shown in Table 1. The mean age in the model training dataset was 30.64 years (SD = 4.84), and 377 blood transfusions (3.72%) were performed in 10,147 operations. 145 transfusions (4.45%) out of 3,110 operations in the simulated training dataset.

**Table 1:** Basic information of patients undergoing cesarean section(n(%) / M(SD))

Variable	Category	Training set N = 10,147	Prediction set N = 3,225
Age(y)	/	30.64(4.84)	31.00(4.24)
Height(cm)	/	160.54(5.47)	160.49(4.89)
Weight(kg)	/	71.99(8.87)	72.22(8.01)
BMI(kg/m^2^)	/	28.18(9.53)	28.16(6.57)
Pre-Hb(g/L)	/	117.54(13.28)	120.22(13.75)
Post-Hb(g/L)	/	101.60(13.38)	105.00(14.39)
RBC(10^12^/L)	/	3.93(0.43)	3.93(0.43)
PLT(10^9^/L)	/	190.32(62.71)	185.22(56.79)
INR	/	0.94(0.12)	0.93(0.08)
PT(s)	/	12.13(1.23)	11.26(1.03)
Surgical levels	4th	166(1.64)	48(1.48)
	3rd	4,284(42.22)	1,643(50.49)
	2nd	5,386(53.08)	1,498(46.04)
	1st	6(0.06)	0(0)
	Null	305(3.01)	65(2.00)
Blood Transfusion	Y	377(3.72)	145(4.45)
	N	9,770(96.28)	3,110(95.55)
Mean Blood Volume/U	/	0.13	0.13

Mean Blood volume is related to Red Blood Cell transfusions, calculated by dividing the volume of RBC transfusions by the total number of surgical procedures. Surgical levels range from first to fourth, with higher levels indicating higher risks, more complex procedures, and greater difficulty.

### Model building and validation

From 2014 to 2019, 14,849 cases of cesarean section operations with blood application were selected and 10,147 cases were screened for model training after passing the admission and discharge criteria. 80% and 8,115 cases were selected as the training set, and 20% and 2,032 cases were used as the test set.

**Fig. 1:**
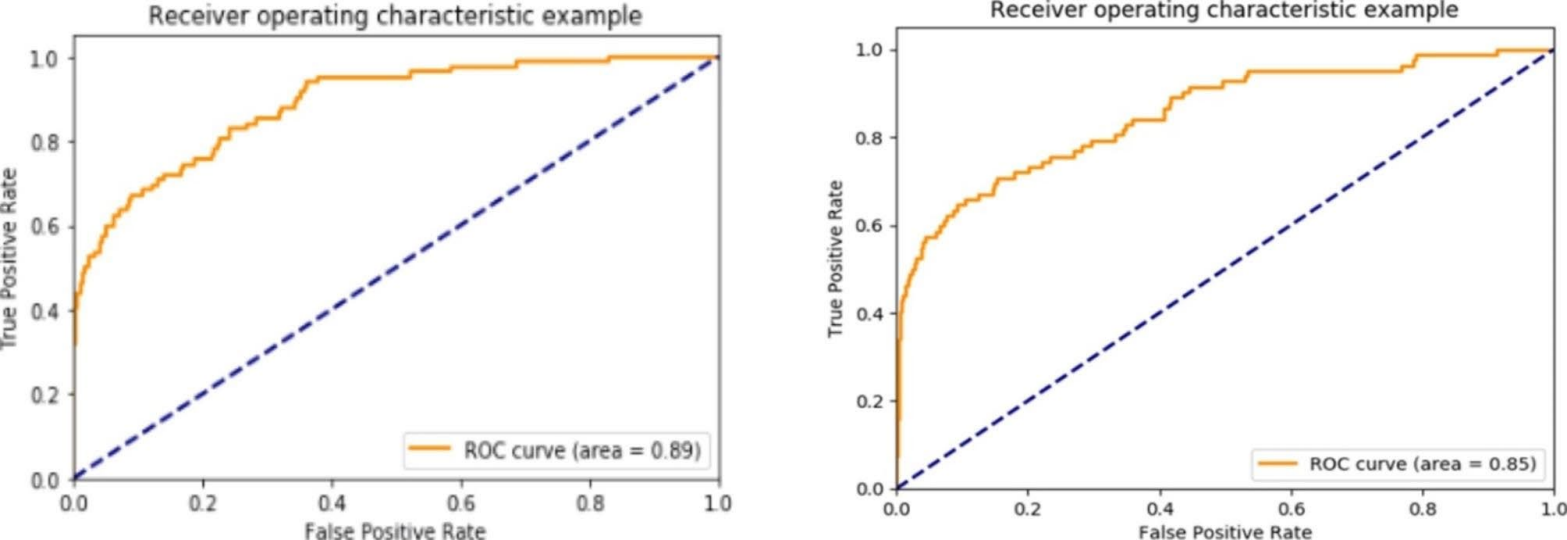
ROC curve for blood transfusion prediction, AI vs logistic regression model

The ROC curve of the model on the test set is shown in Fig. 1, and the area under the AUC = 0.89 better than the multiple regression method (AUC = 0.95). The accuracy of the model for blood transfusion was 96.85%. The statistical standard for the accuracy of the model blood transfusion volume is the calculation of 1U absolute error, the accuracy rate is 86.56%, and the accuracy rate of the blood transfusion population is 45%, higher than the multiple regression method (26.82%).

**Fig. 2:**
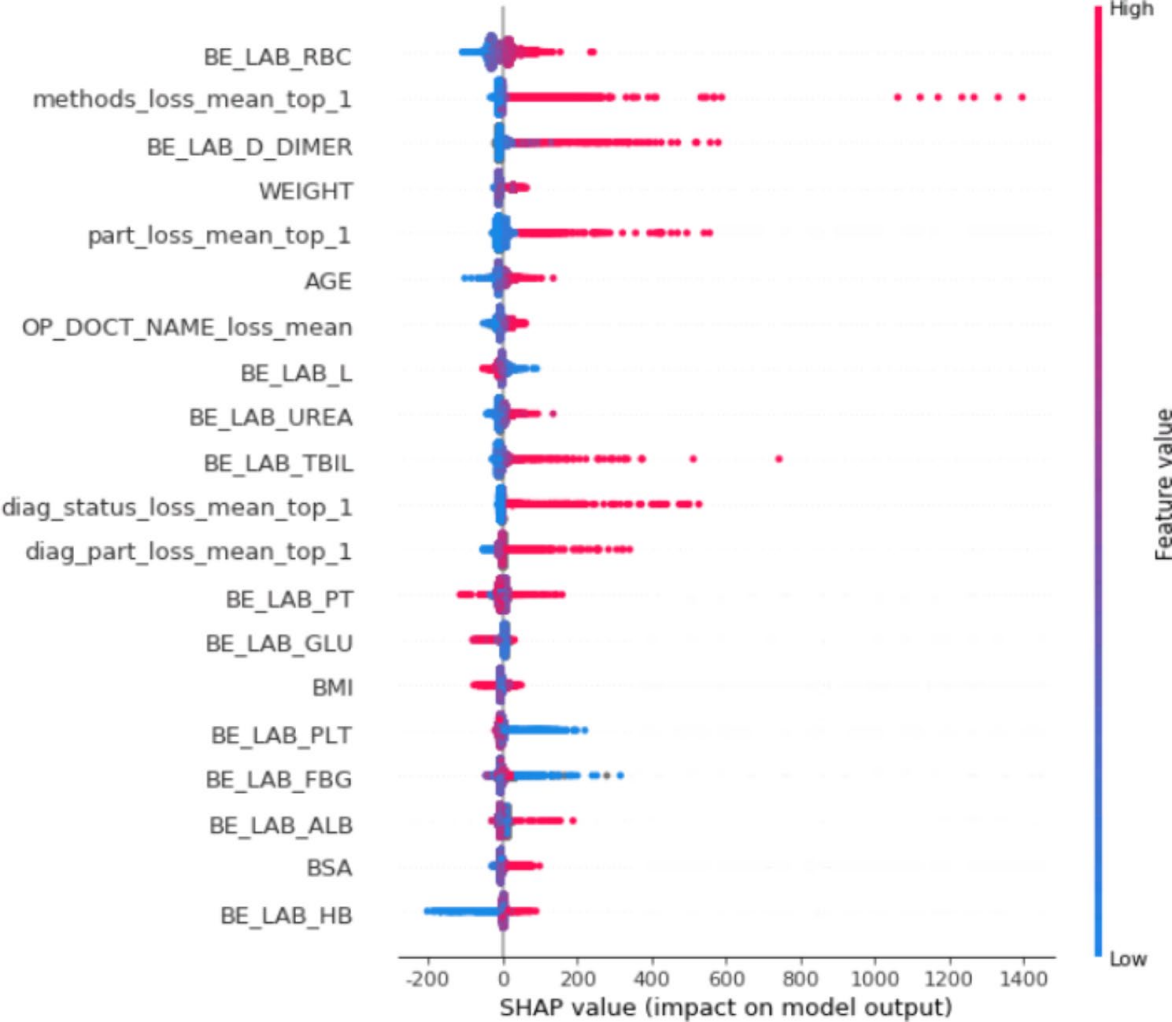
Plots of the importance of the variables and the SHAP variable. The red dots represent large values and the blue dots low values. The SHAP value corresponding to the plot represents the impact on the predict result. The variables are sorted by importance for predicting the likelihood of blood transfusion from high to low

Further feature importance analysis found that preoperative RBCs, surgical methods, surgical sites, and coagulation-related indicators ranked high (Fig. 2).

Our methods can extract complex patterns and correlations from large-scale data. In contrast, multiple regression methods are usually based on statistical models of linear relationships and may perform weakly for complex nonlinear relationships. In terms of blood transfusion prediction, our method can analyze multiple factors, such as patients’ clinical data, physiological indicators, and disease characteristics, and consider them together for comprehensive prediction and decision-making, as well as being able to perform personalized risk factor analysis for individuals. This ability to consider them together and model them non-linearly allows our methods to identify which patients need blood transfusions more accurately, thus improving the accuracy of predictions.

### Simulation prediction effect evaluation

The cesarean section data from January 2020 to September 2021 were selected for simulation prediction, and the blood transfusion volume was evaluated with the target of postoperative Hb of 90 g/L (80 g/L-100 g/L). Compare the forecast results with the actual results to evaluate the forecast effect.

### Blood transfusion or not

In terms of blood transfusion, 92.32% of the predicted and actual blood transfusions were transfused in cesarean section, 63.45% in blood transfusion operations, and 93.67% in non-transfusion operations. 92 of the 289 surgeries predicted blood transfusion were transfused (31.82%), and 2,913 surgeries without blood transfusion were predicted to not receive blood (93.67%). See Table 2 for details.

**Table 2:** Comparison of blood transfusion prediction results

blood transfusion	Predict	Y		Predict	N		Total
	n	%		n	%		n
**Y**	92	63.45		53	36.55		145
**N**	197	6.33		2,913	93.67		3,110
**Total**	289	8.88		2,966	91.12		3,255

### Blood transfusion volume

In terms of blood transfusion volume comparison, among the 3,225 cesarean sections, the actual and predicted blood volume was equal to 2,942 (90.38%). Among them, 29 (20.00%) of 145 blood transfusion operations used the same amount of blood. Although the proportion of model prediction and actual blood use is small in the actual blood use operation, this study further stratified the relevant indicators according to whether blood transfusion and whether the blood use volume was equal (see Table 3 for details). The mean postoperative Hb with equal blood was closer to the predicted target of 90 g/L, the mean postoperative Hb of actual blood transfusion but unequal blood consumption was higher than 90 g/L, and the predicted blood transfusion volume was lower than the actual blood transfusion volume. It is suggested that the predicted blood transfusion volume in some operations is more reasonable than the actual blood transfusion volume in the actual blood transfusion, but the blood use is not equal. Therefore, to evaluate the performance of the model more accurately, it is necessary to further evaluate the relationship between the actual blood transfusion volume and the predicted blood transfusion volume with the postoperative Hb as the target.

**Table 3:** Blood transfusion volume

Blood transfusion	Volume	n	Post-Hb	Used/U	Predict/U
Y	Equal	29	89.10(12.90)	2.00	2.00
	Unequal	116	95.18(17.92)	3.14	1.25
	Total	145	93.97(17.17)	2.91	1.40
N	Equal	2,913	107.26(13.49)	0.00	0.00
	Unequal	197	91.77(13.69)	0.00	2.00
	Total	3,110	106.28(14.01)	0.00	0.13
Total		3,255	105.73(14.39)	0.13	0.18

### Targeting postoperative hemoglobin

First, according to the evaluation method, the actual blood transfusion was evaluated according to the actual postoperative hemoglobin, and the results are shown in Table 4. In blood transfusion surgeries, the accuracy rate of actual blood usage matching AI-predicted blood usage is 56.17% for equal quantities and 50% for unequal quantities. In non-blood transfusion surgeries, the accuracy rate of actual blood usage matching AI-predicted blood usage is 97.94% for equal quantities and 82.23% for unequal quantities.

**Table 4:** Blood transfusion evaluation

blood transfusion	Evaluation			Rate/%	Total
	**I**	**S**	**E**		
**Y**	26	**74**	45	51.03	145
**N**	95	**3,015**		96.95	3,110
**Total**	121	3,089	45	94.90	3,255

Further, the evaluation results of actual blood consumption were calculated according to whether blood was transfused and whether the blood consumption was equal, as shown in Table 5. The accuracy rate of equal blood transfusion was 56.17%, the accuracy rate of unequal blood transfusion was 50.00%; the accuracy rate of unequal blood transfusion was 97.94%; the accuracy rate of unequal blood transfusion was 82.23%.

**Table 5:** Blood transfusion volume evaluation

blood transfusion	Volume	Correct			Wrong	
		**n**	**%**		**n**	**%**
Y	Equal	16	55.17		13	44.83
	Unequal	58	50.00		58	50.00
	Total	74	51.03		71	48.97
N	Equal	2,853	97.94		60	2.06
	Unequal	162	82.23		35	17.77
	Total	3,015	96.95		95	3.05
Total		3,089	94.90		166	5.10

According to the comparison results of actual blood transfusion and predicted blood transfusion, we further performed an in-depth analysis (Table 6). The mean values of preoperative hemoglobin (117.77 g/L), RBC (3.85), and HCT (0.35) were higher in cases where actual blood transfusion occurred but were not predicted. Additionally, the mean postoperative Hb after actual blood transfusion slightly deviated from the target range of 90 g/L (80–100 g/L). Although the preoperative prediction did not match the actual blood transfusion in cases where no blood transfusion was predicted, it still has some clinical reference value. In situations where no blood transfusion was performed, but it was predicted to be, the preoperative Hb (101.49 g/L), RBC (3.53), and HCT (0.31) values were lower. This indicates a higher risk of blood transfusion based on these indicators, making the preoperative prediction clinically relevant. Furthermore, although the predicted amount of blood transfusion is not equal to the actual amount, there is little deviation between the predicted and actual volume of blood transfusion, which still has clinical reference value. However, further optimization can be explored for this particular subset of data in future studies.

**Table 6:** Comparison between different groups

		transfusion				No transfusion	Total
	Pred-Y		Pred-N		Pred-Y	Pred-N	
	Equal n = 29	Unequal n = 63	n = 53		n = 197	n = 2,913	
Pre-Hb	92.31(12.44)	92.08(18.34)	117.77(14.80)		101.49(15.45)	122.42(11.36)	120.22(13.75)
Post-Hb	89.10(12.90)	90.68(14.14)	100.53(20.47)		91.70(13.69)	107.26(13.49)	105.73(14.39)
HCT	0.29(0.04)	0.28(0.05)	0.35(0.04)		0.31(0.04)	0.37(0.03)	0.36(0.04)
Age	31.69(4.38)	31.52(4.97)	31.58(4.87)		32.05(4.51)	30.90(4.18)	31.00(4.24)
Weight	70.54(6.66)	70.15(9.38)	72.66(8.19)		71.83(7.94)	72.30(7.99)	72.22(8.01)
Height	159.68(3.12)	159.44(4.66)	160.71(3.78)		160.60(4.36)	160.51(4.96)	160.49(4.89)
BMI	27.67(2.52)	27.59(3.40)	28.12(2.94)		27.84(2.74)	28.20(6.87)	28.16(6.57)
PLT	190.14(114.76)	154.02(74.20)	181.08(63.14)		176.14(70.83)	186.53(54.05)	185.22(56.79)
RBC	3.44(0.58)	3.11(0.63)	3.85(0.48)		3.53(0.51)	3.98(0.37)	3.93(0.43)
PT	11.53(0.79)	11.66(1.29)	11.46(1.06)		11.33(0.97)	11.24(1.03)	11.26(1.03)
APTT	28.94(3.71)	29.15(4.54)	28.94(3.28)		28.80(3.92)	28.86(3.33)	28.86(3.39)
INR	0.96(0.05)	0.96(0.09)	0.93(0.07)		0.95(0.08)	0.93(0.07)	0.93(0.08)
Apply	3.41(1.64)	3.47(1.50)	3.19(1.23)		2.33(0.79)	2.13(0.52)	2.19(0.66)
Volume	2.00(0.00)	3.38(1.38)	2.85(1.63)		0.00(0.00)	0.00(0.00)	0.13(0.67)
Predict	2.00(0.00)	2.29(0.40)	0.00(0.00)		2.00(0.26)	0.00(0.00)	0.18(0.59)

## Discussion

This study retrospectively analyzed the intraoperative blood transfusion of cesarean section women, used machine learning algorithms to predict intraoperative blood transfusion, and compared the difference between model prediction and actual blood use, to assess the risk of intraoperative blood transfusion for cesarean section women, and forecasts provide data support. There are few previous studies on the prediction of cesarean section blood transfusion, and only about 4% of the actual blood transfusion occurs in the current blood preparation operation. Therefore, this study has important clinical significance and value to develop an artificial intelligence-based model for red blood cell consumption in cesarean section.

Previous studies on the prediction of blood transfusion in the perioperative period of cesarean section mostly used the traditional logistic regression method for risk factor analysis, and seldom specifically predicted blood transfusion. The clinical indicators of prediction are usually limited, and various measurement data such as hematological indicators are rarely included. The sensitivity and specificity of prediction are between 70% and 80%, and the AUC is also low (mostly 0.80–0.90). In this study, machine learning algorithm was used to incorporate multiple clinical indicators related to perioperative blood transfusion, as well as intraoperative related factors such as surgical site and surgical method. Through the verification of test samples, the evaluation index AUC = 0.89. The accuracy rate of blood transfusion is 96.85%, and the accuracy rate of blood transfusion volume within 1U error is 86.56%. The overall performance of the model is high, but the accuracy rate of blood transfusion volume for blood transfusion population is low. In the prediction model constructed by the AI algorithm, the importance of features such as preoperative RBC, surgical method, surgical site, and coagulation-related indicators ranked high. The above variables may be important predictors of blood transfusion during cesarean section.

During the simulation and prediction process, we found that 93.67% of the patients without blood transfusion were predicted to be the same as the actual ones, with a high accuracy rate. In the blood transfusion surgery, the same proportion of predicted blood transfusions was 63.45%, but only 20.00% of the patients had the same blood transfusion volume. The model still needs to be further refined to improve the prediction performance for actual blood transfusion cesarean section, especially the prediction of blood transfusion volume. However, further analysis found that the accuracy rate of actual blood transfusion for cesarean section was 51.03%, and the accuracy rate of no blood transfusion was 96.95%. The accuracy rate of doctors in actual blood use was also relatively low, and there may be insufficient blood use and excessive blood use. Therefore, a direct comparison of model predictions with actual blood consumption may be an underestimate.

In addition, in-depth analysis by grouping according to the matching between predicted results and actual results found that the mean preoperative and postoperative Hb values of patients who were predicted not to receive blood transfusion but actually received blood transfusion were higher, and the model predicted no blood transfusion to have a certain clinical reference value; of patients evaluated their preoperative related indicators and found that the risk of blood transfusion was higher, and the judgment of blood transfusion before surgery was also of reference significance; in addition, the predicted blood transfusion was actually transfused but the amount of blood was different. Although the dosage varies, it can also indicate the risk of blood transfusion. Therefore, from the perspective of preoperative prediction of blood transfusion risk, the model prediction results have great clinical reference value, and as a preoperative evaluation method, it can better provide auxiliary decision support for clinicians.

This study has certain limitations, this is a retrospective clinical study and needs to be further validated in prospective studies. Secondly, this study is a single-center data, and the sample results may be biased, and further multi-center studies are needed to improve the relevant results. Finally, there may be some influencing factors that cannot be obtained from the information system in this study, and potential factors not included may also have some influence on the results.

## Conclusion

In summary, this study used machine learning algorithms to process, analyze and predict the results of a large sample of cesarean section clinical data. The important predictors of blood transfusion during cesarean section included preoperative RBC, surgical method, the site of surgery, coagulation-related indicators, and other factors. At the same time, the AI model was compared with the actual blood consumption, and it was found that the overall accuracy of the AI model was higher. Although the prediction of blood transfusion volume has a low degree of matching with the actual blood use, from the perspective of preoperative identification of high risk of blood transfusion, the results of model prediction can provide a good decision support for preoperative evaluation of obstetric cesarean section, and then promote the realization of accurate perioperative blood management of obstetric cesarean section patients.

## Data Availability

The datasets generated and analyzed during the current study are not publicly available due to hospital data confidentiality requirements but are available from the corresponding author on reasonable request.
